# The herpesvirus 8 encoded chemokines vCCL2 (vMIP-II) and vCCL3 (vMIP-III) target the human but not the murine lymphotactin receptor

**DOI:** 10.1186/1743-422X-5-50

**Published:** 2008-04-21

**Authors:** Hans R Lüttichau

**Affiliations:** 1 Laboratory for Molecular Pharmacology, Department of Neuroscience and Pharmacology, Panum Institute, DK-2200 Copenhagen, Denmark; 2 Department for Infectious Diseases, Hvidovre Hospital Copenhagen, Denmark

## Abstract

**Background:**

Large DNA-viruses such as herpesvirus and poxvirus encode proteins that target and exploit the chemokine system of their host. The Kaposi sarcoma- associated herpes virus (KSHV) encodes three chemokines. Two of these, vCCL2 and vCCL3, target the human lymphotactin receptor as an antagonist and a selective agonist, respectively. Therefore these virally endcoded chemokines have the potential to be used as tools in the study of lymphotactin receptor pathways in murine models.

**Results:**

The activities of vCCL2, vCCL3, human lymphotactin (XCL1) and murine lymphotactin (mXCL1) were probed in parallel on the human and murine lymphotactin receptor (XCR1 and mXCR1) using a phosphatidyl-inositol assay. On the human XCR1, vCCL3, mXCL1 and XCL1 acted as agonists. In contrast, only mXCL1 was able to activate the murine lymphotactin receptor. Using the same assay, vCCL2 was able to block the response using any of the three agonists on the humane lymphotactin receptor with IC_50_s of 2–3 nM. However, vCCL2 was unable to block the response of mXCL1 through the murine lymphotactin receptor.

**Conclusion:**

This study shows that vCCL2 and vCCL3 cannot be used to investigate lymphotactin receptor pathways in murine models. These results also add vCCL2 and vCCL3 to a growing list of viral chemokines with known human chemokine receptor targets, which do not target the corresponding murine receptors. This fits with the observation that viral and endogenous ligands for the same human chemokine receptor tend to have relatively divergent amino-acid sequences, suggesting that these viruses have fine-tuned the design of their chemokines such that the action of the viral encoded chemokines cannot be expected to cross species barriers.

## Background

During the last 15 years, more than 40 chemokines have been identified in the human genome and nearly all have been characterized pharmacologically as agonists and led to the identification of 18 signaling 7TM chemokine receptors [1,2].

Chemokines are 70–80 amino acid proteins with a characteric three-dimensional fold, which are involved in guiding and activating distinct leukocyte subsets. Chemokines can be divided into four sub-families on the basis of the pattern and number of the conserved cysteine residues located near their N-terminus, which are involved in disulfide binding formation; the CC-, CXC-, CX_3_C and XC family, respectively. The XC-chemokines have only one cysteine in the N-terminus. Chemokines act through 7TM GPCRs of which we today know ten CC-chemokine receptors (CCR1-10), six CXC-receptors (CXCR1-6), one CX_3_C-receptor (CX_3_CR1) and one XC-receptor (XCR1). The role played by the lymphotactin receptor (XCR1) in the immune system is poorly understood.

In the same period, seven chemokines encoded by large human DNA viruses have been found by genomic sequence analysis. Most of these have been characterized and have been found to have different pharmacological phenotypes as some target multiple receptors, some only one receptor, some act as agonists, while others act as antagonists [3-11](Table 1).

**Table 1:** Chemokines encoded by human viruses and their human and murine chemokine receptor targets.

**Virus**	**Gene**	**Protein**	**human chemokine receptor targets**	**Ref**	**also targeting the corresponding murine chemokine receptor**	**Ref**
**CMV**	UL146	vCXCL1	**Selective CXCR2 agonist**	11	**No**	20
	UL147	vCXCL2	**?**			
**HHV6**^a^	U83B	vCCL4	**Selective CCR2 agonist**	9	**?**	
**HHV8**	K6	vCCL1 (vMIP-I)	**Selective CCR8 agonist**	3,5,10	**Yes**	18
	K4	vCCL2 (vMIP-II)	**Broadspectrum chemokine receptor**			
			**antagonist**			
			**CCR1**	6,7	**Yes**	19
			**CCR2**	6,7	**Yes**	19
			**CCR5**	6,7	**Yes**	19
			**XCR1**	7	**No**	This paper
			**CX3CR1**	7	**?**	
			**CXCR4**	6,7	**?**	
			**CCR3 agonist**	17	**?**	
	K4.1	vCCL3 (vMIP-III)	**Selective XCR1 agonist**	10	**No**	This paper
**MCV**	MC148	MCC	**Selective CCR8 antagonist**	7	**No**	18

^a^The protein product from the U83 gene of HHV6A has not been included as its signal sequence include a premature stop codon.

Obviously viral encoded chemokines are important in the study of viral pathogenesis, but they can also be used as tools in the investigation of specific chemokine receptors. Blocking of chemokine receptor action is important in several assays and immunological models studying the chemokine system. One example is the selective CCR8 antagonist MC148 encoded by the *Molluscuum Contagiosum Virus *[7]. Another example is the broad-spectrum chemokine antagonist vCCL2 encoded by HHV8, which blocks a number of chemokine receptors such as CCR1, CCR2, CCR5, CX3CR1, CXCR4 and the lymphotactin receptor XCR1 [6,7]. Thus vCCL2 has been shown to reduce the inflammatory response in small animal models models [12-15]. However, viral-encoded agonists are also useful in the investigation of the role of a chemokine receptor even when the endogenous human ligand has been identified, because they can have greater potency and be more stable than their human counterparts. This is the case for another HHV8 encoded chemokine vCCL3, which was recently found to have a 10-fold higher potency than lymphotactin on the human lymphotactin receptor [10].

**Figure 1:**
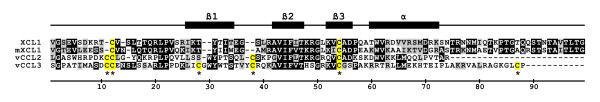
**Alignment of human, murine and viral ligands for the human and the murine lymphotactin receptor**. The upper panel shows the primary structure of the two HHV8 encoded chemokines, vCCL2, vCCL3, the human lymphotactin XCL1 and the murine lymphotactin mXCL1 aligned using CLUSTALW from Kyoto University Bioinformatics Center. Identical amino acids are shown in white on black, whereas similar amino acids are shown in black on light grey. Cysteines are shown in black on yellow and presumed disulfide bridges are marked with an asterisk. Likely O-glycosylation sites using the CBS prediction server are marked white on blue. The secondary structure of XCL1 as determined by NMR is indicated by the line above the alignment [21].

Thus HHV8 encodes both the only known high-affinty lymphotactin receptor antagonist, vCCL2, as well as the most potent agonist, vCCL3, known to XCR1 (Figure 1). Therefore both vCCL2 and vCCL3 are valuable tools in evaluating the role of the lymphotactin receptor. However, when using animal models it is obviously important to characterize these proteins on the specific animal chemokine receptors. Here we report the characterization of the viral-encoded proteins vCCL2 and vCCL3 on the murine lymphotactin receptor done in parallel with the humane XCR1.

## Results and discussion

Recently, we reported that the HHV8 encoded chemokine vCCL3 is a selective agonist of the human lymphotactin receptor XCR1, while vCCL2, encoded by the same virus, acts as an antagonist on this same receptor [10].

### Agonistic activity on XCR1 and mXCR1

**Figure 2:**
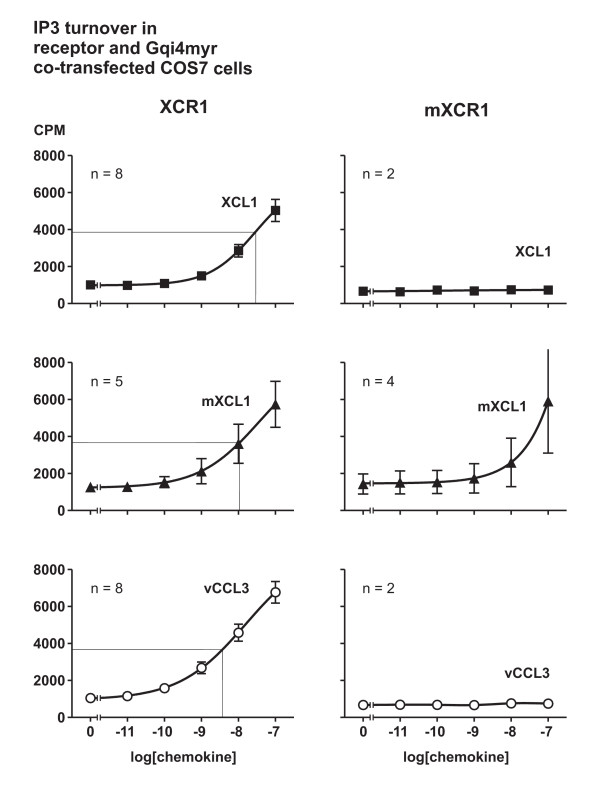
**Activation of the human and murine lymphotactin receptor by human, murine and viral agonists**. Dose-response experiments measuring IP_3 _turnover in COS-7 cells transiently transfected with the human and murine lymphotactin receptors XCR1 and mXCR1 and the promiscuous chimeric G-protein Gqi4myr using increasing concentrations of the agonists XCL1 (black square), mXCL1 (black triangle) and vCCL3 (white circle). The thin line indicate the EC_50 _for the particular ligand (assuming Emax equal to that of vCCL3). All assays were performed in duplicate.

In order to determine whether these two chemokines encoded by a human virus and acting on a human receptor also were able to target the corresponding murine receptor we performed phosphatidyl-inositol assays using a promiscuous chimeric G-protein[16] co-transfected with either the human XCR1 gene inserted in the pcDNA3.1 vector or the murine XCR1 gene inserted into the pTEJ vector. As expected, XCL1, mXCL1 and vCCL3 activated the human lymphotactin receptor in a dose responsive manner (Figure 2). As reported earlier [10], the potency of vCCL3 (EC_50 _= 3.7 nM) was nearly 10-fold greater than the potency of XCL1 (EC_50 _= 30 nM, assuming Emax equal to that of vCCL3) on the human XCR1 receptor. Interestingly, murine lymphotactin had a 3-fold higher potency (EC^50 ^= 11 nM) than human lymphotactin on the human lymphotactin receptor.

In contrast, only mXCL1 was able to activate the murine lymphotactin receptor, while vCCL3 and XCL1 were unable to do so (Figure 2). It should be noted that only a relatively high concentration of mXCL1 (100 nM) consistently generated an IP3 response through the murine lymphotactin receptor. This observation could be explained if mXCL1 was not properly folded or was partly proteolyzed. However, this is unlikely as 1 nM of mXCL1 in all assays was able to activate the human lymphotactin receptor. To rule out a vector-specific effect, we performed similar assays using the mXCR1 gene inserted into pcDNA3.1. However, no difference in results was found using the two vector constructs (data not shown). The low potency of mXCL1 on the mXCR1 in the IP3 assay could also be related to the cell-line used, but again this explanation seems unlikely as we also had difficulties in generating calcium mobilization responses to mXCL1 in single clones of L1.2- and 300.19- cells transfected with murine lymphotactin receptor cDNA, which suggested that mXCL1 indeed had low potency for the lymphotactin receptor. A more likely explanation of the poor potency of mXCL1 for mXCR1 could be that either the extra N-terminal methionine or the lack of glycosylation of the E. Coli-produced recombinant murine lymphotactin prevented proper activation of the murine but not the human lymphotactin receptor.

### Antagonistic activity on XCR1 and mXCR1

**Figure 3:**
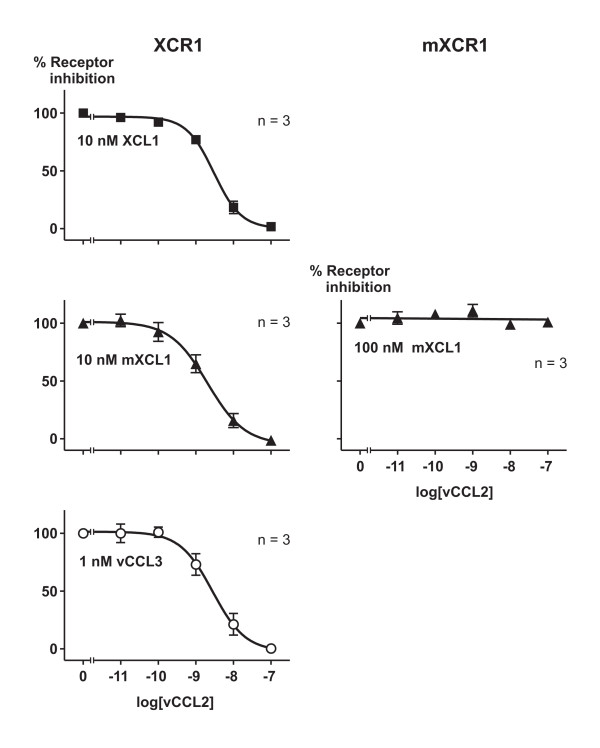
**Inhibition of the human and murine lymphotactin receptor by the viral antagonist vCCL2**. Dose-response experiments for inhibition of IP_3_-turnover by the antagonist vCCL2 induced by XCL1 (black square) mXCL1 (black triangle) and vCCL3 (white circle) in COS-7 cells transiently transfected with the human and murine lymphotactin receptors XCR1 and mXCR1 and the promiscuous chimeric G-protein Gqi4myr. All assays were performed in duplicate.

We next tested whether vCCL2 was able to act as an antagonist on the murine and the human lymphotactin receptors. As reported before [10], vCCL2 did block responses through XCR1 using submaximal doses of either XCL1, mXCL1 or vCCL3 (Figure 3). As expected the IC_50 values _were almost the same no matter which agonist was used (mXCL1 had an IC_50 _= 2.1 nM; XCL1 had an IC_50 _= 3.0 nM and vCCL3 had an IC_50 _= 2.8 nM). In contrast, vCCL2 was unable to inhibit the response of mXCL1 through the murine lymphotactin receptor (Figure 3).

### Virally encoded chemokines and species barriers

**Figure 4:**
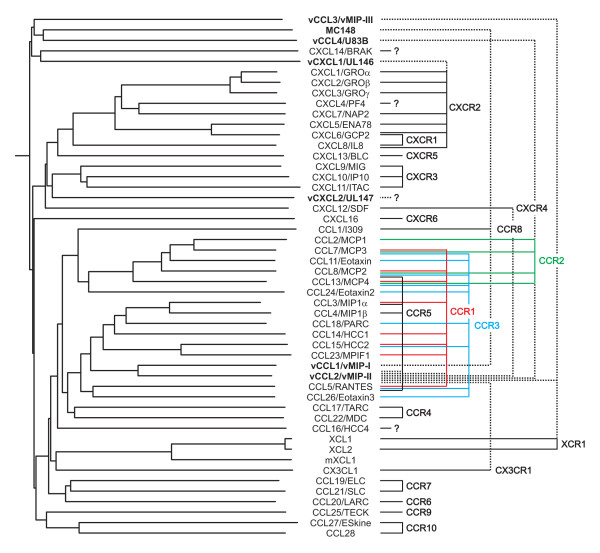
**Amino-acid sequence similarity of human and viral encoded chemokines compared to their chemokine receptor targets**. Left side of the figure is a comparison of the similiarity of the primary protein sequence of human and virally encoded chemokines (using ClustalW 1.83 and an unbranched dendrogram) with the receptor targets of the specific chemokines seen on the right side of the figure. The names of the virally encoded chemokines are highlighted in bold. On the right hand side a line is used to illustrate that a specific chemokine is a ligand for a specific chemokine receptor. The line is unbroken for endogenous human chemokines and dotted for virally encoded chemokines. The murine lymphotactin has been included to illustrate the great similarity between XCL1 and mXCL1.

Surprisingly, XCL1 could not activate the murine lymphotactin receptor although the sequences of mXCL1 and XCL1 are very similar (60 % identity and 84 % similarity using BLASTP 2.2.17 at the NCBI website)(Figure 1). In contrast, vCCL2 and vCCL3 have only 31 and 32 % identity and 50 and 54 % similarity to XCL1, although all three ligands target the same receptor. These points are illustrated on the left side of Figure 4. XCL1 and mXCL1 are clustered, whereas vCCL2 and vCCL3 are more distantly related to XCL1 although they target the same receptor (right side of Figure 4). As can be seen from Figure 4, it seems to be a rule that human encoded chemokines acting on a particular chemokine receptor, cluster with each other but not with the viral encoded chemokines targeting the same receptor. There is one exception, vCCL2, which on the dendrogram in Figure 4 lies in the middle of a cluster of CCR1, CCR2, and CCR5 ligands. However, vCCL2 also targets XCR1, CX3CR1 and CXCR4 and it does not cluster with the ligands of these receptors.

This sequence divergence between viral and human chemokines may explain why human and not viral encoded chemokines in general are able to cross a species barrier. Human encoded chemokines are very similar to their murine counterparts but very dissimilar to the viral encoded chemokines they share receptor targets with. Thus one can imagine that a virus during evolution has picked up an ancestral chemokine gene and optimized it in a unique way to suit the virus, while retaining the ability to target the corresponding chemokine receptor of its host. However, during this process the hijacked chemokine has changed so much that it has lost the ability to cross species barriers. As seen in Table 1, the CMV encoded chemokine vCXCL1, the pox virus encoded chemokine MC148 and, as shown in this paper, the KSHV encoded chemokines vCCL2 and vCCL3 are not able to target their corresponding murine receptors, while only vCCL1 encoded by KSHV has retained this ability [3,5-7,10,11,17-20].

vCCL2 has been used in several *in vivo *models, especially in rodent models, as a broad-spectrum chemokine receptor blocker. For example vCCL2 has been shown to reduce T-cell mediated inflammation in a murine lymphocytic choriomeningitis model [14], to protect the brain of mice against cerebral ischemia [15], to reduce inflammation in a rat model of spinal cord contusion [13] and in a rat model of glomerulonephritis [12]. The results from this study suggest that the reduction of inflammation by vCCL2 in these experiments is not due to inhibition of the lymphotactin receptor, but must be due to inhibition of one or more of the CCR1, CCR2, CCR5, CX3CR1 and CXCR4 receptors.

## Conclusion

The chemokines vCCL2 and vCCL3 encoded by KSHV do not target the murine lymphotactin receptor, although they act as a high potency antagonist and agonist respectively, on the human lymphotactin receptor. So the only tool left, for investigating the role of the lymphotactin receptor pathways in murine models, is the commercially available recombinant form of murine lymphotactin with a rather low potency on the mXCR1 receptor. Furthermore, when the findings of this paper are combined with the results from other studies on the ability of chemokines encoded by human viruses to target the murine counterparts to their human chemokine receptor targets, it can be concluded that they rarely do so. In contrast, human encoded chemokines in general are able to cross the species barrier and target their corresponding murine chemokine receptors.

## Methods

### Chemokines

mXCL1, XCL1, vCCL2 were purchased from R&D (Minneapolis, MN). Recombinant vCCL3 (GenBank accession number U93872) was produced as described previously [10], briefly cell media collected from COS7 cells transfected with the K4.1 gene from HHV8 was collected and purified on a cation-exchange column followed by reverse phase HPLC. The elution position of the recombinant vCCL3 protein as well as the purity was identified with mass-spectroscopy and NH_2_-terminal sequence analysis on an ABI 494 protein sequencer (Perkin-Elmer, CA).

### Cloning of mXCR1

The mXCR1 gene was amplified by PCR from a murine cDNA library and inserted into the pcDNA3.1 vector and the pTEJ8 vector. Start- and end-primers were designed from the GenBank accession number NM 011798. Nucleotide sequence analysis was performed on an ABI 310 sequence system (Perkin-Elmer, CA) in-house or by MWG Biotech (Ebersberg, Germany). The human XCR1 gene inserted in the pcDNA3.1 vector was purchased from the UMR cDNA Resource Center (Rolla, MO).

### Stable cell lines

mXCR1 in pTej was transfected into the murine pre-B cell line L1.2 and hXCR1 in pcDNA 3.1 was transfected into the murine pre-B cell line 300.19. Stable transfectants were obtained after limiting dilution and chemical selection with G418 and functional clones were selected based upon their calcium responses to mXCL1 and XCL1, respectively.

### Phosphatidyl-inositol assay

COS-7 cells were transiently transfected by a calcium phosphate precipitate method with addition of chloroquine. Briefly, 2 × 10^6 ^COS-7 cells were transfected with 30 ug of cDNA encoding the promiscuous chimeric G-protein, GαΔ6qi4myr (abbreviated as Gqi4myr), which allows the Gαi-coupled receptor to couple to the Gαq pathway (phospholipase C activation measured as PI-turnover) [16], with or without 20 ug receptor (mXCR1 or XCR1) cDNA. After transfection, COS-7 cells (2.5 × 10^4 ^cells/well) were incubated for 24 hours with 2 μCi of ^3^H-*myo*-inositol in 0.4 ml growth medium per well in 24-multiwells tissue culture plates. Cells were washed twice in 20 mM Hepes, pH 7.4, supplemented with 140 mM NaCl, 5 mM KCl, 1 mM MgSO_4_, 1 mM CaCl_2_, 10 mM glucose and 0.05% (w/v) bovine serum albumin and were incubated in 0.4 ml of the same buffer supplemented with 10 mM LiCl at 37°C for 15 minutes. The ligands were subsequently added and incubated for 90 min. In the antagonist assay vCCL2 was added 10 min before the agonist to ensure proper interaction of the receptors with vCCL2. Cells were extracted by addition of 1 ml 10 mM Formic acid to each well followed by incubation on ice for 30–60 min. The generated [^3^H]-inositol phosphates were purified on AG1-X8 anion-exchange resin (Bio-Rad Laboratories, Hercules, CA). Determinations were made in duplicate.

### Calcium mobilization experiments

L1.2 cells stably transfected with mXCR1 were loaded with Fura-2AM (Molecular Probes, Eugene, OR) in RPMI with 1% FCS for 20–30 min. and washed in the same buffer. Aliqouts were made of 1 × 10^6 ^cells, each aliqout was pelleted and resuspended in 500 ul PBS 1% FCS with 10 mM EGTA. Flourescence was measured on a Jobin Yvon FlouroMax-2 (Jobin Yvon Spex, Cedex, France) as the ratio of emission at 490 nm when excited at 340 nm and 380 nm respectively.

## Abbreviations

KSHV, Kaposi sarcoma-associated herpes virus; GPCR, G-protein-coupled receptor; HHV8, human herpesvirus 8; IP3, inositol-tri-phosphate; vMIP, viral macrophage inflammatory protein; XCL, lymphotactin; XCR1 lymphotactin receptor; 7TM, 7 transmembrane.

## Competing interests

The author declares that they have no competing interests.

## Authors' contributions

HRL is responsible for all aspects of this article.

## References

[B1] Murphy PM, Baggiolini M, Charo IF, Hebert CA, Horuk R, Matsushima K, Miller LH, Oppenheim JJ, Power CA (2000). International union of pharmacology. XXII. Nomenclature for chemokine receptors. Pharmacol Rev.

[B2] Rollins BJ (1997). Chemokines. Blood.

[B3] Dairaghi DJ, Fan RA, McMaster BE, Hanley MR, Schall TJ (1999). HHV8-encoded vMIP-I selectively engages chemokine receptor CCR8. Agonist and antagonist profiles of viral chemokines. J Biol Chem.

[B4] Dewin DR, Catusse J, Gompels UA (2006). Identification and characterization of U83A viral chemokine, a broad and potent beta-chemokine agonist for human CCRs with unique selectivity and inhibition by spliced isoform. J Immunol.

[B5] Endres MJ, Garlisi CG, Xiao H, Shan L, Hedrick JA (1999). The Kaposi's sarcoma-related herpesvirus (KSHV)-encoded chemokine vMIP-I is a specific agonist for the CC chemokine receptor (CCR)8. J Exp Med.

[B6] Kledal TN, Rosenkilde MM, Coulin F, Simmons G, Johnsen AH, Alouani S, Power CA, Luttichau HR, Gerstoft J, Clapham PR, Clark-Lewis I, Wells TN, Schwartz TW (1997). A broad-spectrum chemokine antagonist encoded by Kaposi's sarcoma-associated herpesvirus. Science.

[B7] Luttichau HR, Stine J, Boesen TP, Johnsen AH, Chantry D, Gerstoft J, Schwartz TW (2000). A highly selective CC chemokine receptor (CCR)8 antagonist encoded by the poxvirus molluscum contagiosum. J Exp Med.

[B8] Luttichau HR, Lewis IC, Gerstoft J, Schwartz TW (2001). The herpesvirus 8-encoded chemokine vMIP-II, but not the poxvirus-encoded chemokine MC148, inhibits the CCR10 receptor. Eur J Immunol.

[B9] Luttichau HR, Clark-Lewis I, Jensen PO, Moser C, Gerstoft J, Schwartz TW (2003). A highly selective CCR2 chemokine agonist encoded by human herpesvirus 6. J Biol Chem.

[B10] Luttichau HR, Johnsen AH, Jurlander J, Rosenkilde MM, Schwartz TW (2007). Kaposi sarcoma-associated herpes virus targets the lymphotactin receptor with both a broad spectrum antagonist vCCL2 and a highly selective and potent agonist vCCL3. J Biol Chem.

[B11] Penfold ME, Dairaghi DJ, Duke GM, Saederup N, Mocarski ES, Kemble GW, Schall TJ (1999). Cytomegalovirus encodes a potent alpha chemokine. Proc Natl Acad Sci USA.

[B12] Chen S, Bacon KB, Li L, Garcia GE, Xia Y, Lo D, Thompson DA, Siani MA, Yamamoto T, Harrison JK, Feng L (1998). In vivo inhibition of CC and CX3C chemokine-induced leukocyte infiltration and attenuation of glomerulonephritis in Wistar-Kyoto (WKY) rats by vMIP-II. J Exp Med.

[B13] Ghirnikar RS, Lee YL, Eng LF (2000). Chemokine antagonist infusion attenuates cellular infiltration following spinal cord contusion injury in rat. J Neurosci Res.

[B14] Lindow M, Nansen A, Bartholdy C, Stryhn A, Hansen NJ, Boesen TP, Wells TN, Schwartz TW, Thomsen AR (2003). The virus-encoded chemokine vMIP-II inhibits virus-induced Tc1-driven inflammation. J Virol.

[B15] Takami S, Minami M, Nagata I, Namura S, Satoh M (2001). Chemokine receptor antagonist peptide, viral MIP-II, protects the brain against focal cerebral ischemia in mice. J Cereb Blood Flow Metab.

[B16] Kostenis E (2001). Is Galpha16 the optimal tool for fishing ligands of orphan G-protein-coupled receptors?. Trends Pharmacol Sci.

[B17] Boshoff C, Endo Y, Collins PD, Takeuchi Y, Reeves JD, Schweickart VL, Siani MA, Sasaki T, Williams TJ, Gray PW, Moore PS, Chang Y, Weiss RA (1997). Angiogenic and HIV-inhibitory functions of KSHV-encoded chemokines. Science.

[B18] Luttichau HR, Gerstoft J, Schwartz TW (2001). MC148 encoded by human molluscum contagiosum poxvirus is an antagonist for human but not murine CCR8. J Leukoc Biol.

[B19] Rubant S, Ludwig RJ, Pfeffer J, Schulze-Johann P, Kaufmann R, Pfeilschifter JM, Boehncke WH, Radeke HH (2006). Eukaryotic expression of the broad-spectrum chemokine receptor antagonist vMIP-II and its effects on T-cell function in vitro and in vivo. Exp Dermatol.

[B20] Sparer TE, Gosling J, Schall TJ, Mocarski ES (2004). Expression of human CXCR2 in murine neutrophils as a model for assessing cytomegalovirus chemokine vCXCL-1 function in vivo. J Interferon Cytokine Res.

[B21] Kuloglu ES, McCaslin DR, Kitabwalla M, Pauza CD, Markley JL, Volkman BF (2001). Monomeric solution structure of the prototypical 'C' chemokine lymphotactin. Biochemistry.

